# A nosocomial hepatitis viral c transmission in an auto-dialysis center: risk factors using a root-causes analysis

**DOI:** 10.1186/2047-2994-4-S1-P229

**Published:** 2015-06-16

**Authors:** L Haudebourg, E Seringe, M Aggoune, M Bertrand, F Boulot, P Astagneau

**Affiliations:** 1 North France Healthcare Infection control Centre (CClin Paris-Nord), Paris, France; 2 Dialysis Center, France

## Introduction

Hemodialysis is one of healthcare settings where HCV transmissions have been frequently reported.

## Objectives

This work aims to identify the contributing factors of a patient-to-patient HCV transmission in an auto-dialysis centre

## Methods

An investigation was conducted based on observation of healthcare practices and interviews of the nursing staff. A root-causes analysis was performed to identify patent and latent failures involved in HCV transmission using the association of litigation and risk management method (ALARM).

## Results

On the 17^th^ of January 2014, the referral centre for infection control received a report about a case of HCV seroconversion in an auto-dialysis centre in March 2013. The contamination period was estimated to be between October 2012 and February 2013, during which the patient was dialyzed on the same day as another patient known to be infected with HCV. The two viral strains compared by the National Center of Reference for hepatitis were identical.

The main patent failures were: non-optimal compliance with standard precautions, lack of patients’ hand hygiene, blurred job descriptions for hospital cleaning personnel and non-compliance with the safety period between two consecutive dialysis sessions.

Contributing factors were linked to: the patients, poorly trained in hygiene protocols before their arrival at the dialysis center, sometimes aggressive and asking to be quickly connected for dialysis as soon as they arrived; to the professionals, who underestimated the viral risk of splashing and did not audit the patients’ hand hygiene; to the team, the same pair of nurse and hospital cleaning personnel had been providing care to the same, which has contributed to the deterioration of their relationship.

Latent failures were: Institutional, with an inadequate safety culture; Organizational, with a minimal leadership and the absence of job descriptions.

## Conclusion

Clinical management should be better promoted to control viral nosocomial transmission, including team work and safety culture. The root-causes analysis using ALARM is an effective, reproducible and comprehensive approach to identifying deep causes related to institutional, organisational and management factors.

## Disclosure of interest

None declared.

